# 

**Published:** 1889-08

**Authors:** 


### CONTENTS.

ORIGINAL COMMUNICATIONS—

Ten Consecutive Laparotomies. By Virgil O. Hardon, M. D., Atlanta, Ga.......... 323

Correcting the Whole Error of Refraction and the Necessity for the Use of a Mydriatic. By R. O. Cotter, M. D., Macon, Ga.......... 330

Electricity in Gynecology. By W. E. B. Davis, M. D., Birmingham, Ala.......... 337

A Report of Two Incomplete Ovariotomies—One Fatal. By John J. Buchanan, M. D., Pittsburg, Pa.......... 342

Two Unusual Cases of Obstetrics. By H. McHatton, M. D., Macon, Ga.......... 347

Suspension in the Treatment of Affections of the Spinal Cord. By Alexander B. Shaw, M. D., St. Louis, Mo.......... 349

A Case of Gastrostomy. By Jas. McFadden Gaston. M. D., Atlanta, Ga.......... 360

SOCIETY REPORTS—

Allegheny County Medical Society.......... 363

CORRESPONDENCE—

Our New York Letter.......... 368

—A Case of Triplets, by J. D. Westervelt, M. D.......... 372

BOOK REVIEWS—

A Treatise on Surgery, its Principles and Practice. By T. Holmes, M. A. Cantab.......... 373

EDITORIAL—

Salutatory.......... 374

Southern Surgical and Gynecological Association.......... 375

ITEMS—

American Rhinological Association. Vomiting in Pregnancy—Ice Water Enemata in the Treatment of Diarrhœa—Dr. Brown-Séquard's Hypodermic Fluid—Experimental Studies on Hyperpyrexias—Continence and Syphilis—A New Symptom of Pericarditis—Influence of the Pneumogastric Nerves on the Urinary Secretion—Action of Cocaine on the Body Temperature—Brown-Séquard's New Rejuvenator—Dry Chloride of Sodium in the Treatment of Subinvolution of the Uterus—Vermont Microscopical Association—Changes in Faculty of College of Physicians and Surgeons, Baltimore, Md.—Explanation by Dr. J. S. Todd.......... 378, 386

PERSONALS—xxxvi, xxxvii, xxxviii.

